# PD-L1 is highly expressed in ovarian cancer and associated with cancer stem cells populations expressing CD44 and other stem cell markers

**DOI:** 10.1186/s12885-022-10404-x

**Published:** 2023-01-05

**Authors:** Kholoud Alwosaibai, Salmah Aalmri, Miral Mashhour, Salim Ghandorah, Abdulraheem Alshangiti, Faisal Azam, Waleed Selwi, Lubna Gharaibeh, Yasser Alatawi, Zainab Alruwaii, Hashem O. Alsaab

**Affiliations:** 1 Research Center, Biomedical Research Department, King Fahad Specialist Hospital, Dammam, Saudi Arabia; 2 Department of Pathology and Lab Medicine, King Fahad Specialist Hospital, Dammam, Saudi Arabia; 3 Department of Medical Oncology, King Fahad Specialist Hospital-Dammam, Dammam, Saudi Arabia; 4 Pharmacological and Diagnostic Research Center, Faculty of Pharmacy, Al-Ahliyya Amman University, Amman, Jordan; 5 Department of Pharmacy Practice, Faculty of Pharmacy, University of Tabuk, Tabuk, Saudi Arabia; 6 Department of Anatomic Pathology, Dammam Regional Laboratory and Blood Bank, Dammam, Saudi Arabia; 7 Department of Pharmaceutics and Pharmaceutical Technology, Taif University, P.O BOX 11099, Taif, Saudi Arabia

**Keywords:** Ovarian cancer, Immunotherapy, Stem cell markers, Immune-checkpoints markers, PD-L1, CD44

## Abstract

**Background:**

Immune checkpoint inhibitors, including PD-L1 (programmed death ligand-1) inhibitors have well documented anticancer therapeutic effect in most types of cancers but its use in the treatment of ovarian cancer is not yet proven. The aim of our study is to explore the predictive biomarkers in ovarian cancer and its association with the outcomes. We have investigated the role of PD-L1 expressions in the tumor microenvironment cells including immune cells and cancer stem cells in different types of ovarian cancer.

**Methods:**

A total of 119 surgical archived ovarian cancer samples were collected from the pathology department at King Fahad Specialist Hospital, Dammam, Saudi Arabia that included serous carcinomas, clear cell carcinomas, mucinous carcinomas, endometrioid carcinomas, and granulosa cell tumors. Immunohistochemistry (IHC) staining was performed using (i) PD-L1 antibodies to detect PD-L1 expressions; (ii) CD8 and CD4 to detect Tumor Infiltrating Lymphocytes (TILs); and (iii) CD44, LGR5, and ALDH2 to detect stem cell markers. The clinicopathological data were collected from patients’ medical record to investigate the association with PD-L1, TILs, and stem cells expressions.

**Results:**

We report high PD-L1 expressions in 47.8% of ovarian cancer samples. PD-L1 expressions were detected in different types of epithelial ovarian cancer and were not associated with poor prognosis of ovarian cancer. However, determining the expression levels of TILs in the ovarian cancer tissues found that 81% (*n* = 97) of ovarian cancer samples have TILs that express both of CD8 and CD4 and significantly associated with high PD-L1 expressions. Interestingly, we have found that ovarian cancer tissues with high expressions of PD-L1 were associated with high expressions of stem cells expressing CD44 and LGR5.

**Conclusions:**

PD-L1 is highly expressed in the serous type of ovarian carcinomas and the overall expression of PD-L1 is not associated with poor survival rate. Furthermore, PD-L1 expressions are strongly associated with TILs and stem cell markers in ovarian cancer. Inhibiting the PD-L1 using immune checkpoint inhibitors might downregulate stem cell population that known to be associated with cancer recurrence.

## Introduction

Ovarian cancer is still the leading cause of cancer-related mortality among women [1]. It is identified as the most lethal cancer commonly diagnosed among women aged between the ages of 55 and 64 years [2]. The survival rate of women for 5 years or more from the date of diagnosis is 45%. This survival rate is even lower for the patients that were diagnosed at a later stage [2]. The absence of disease symptoms is the main reason for delay in diagnosis, which necessitates more understanding of ovarian cancer. The main stay of treatments for any ovarian cancer stage are surgery and chemotherapy [3]. However, the cumulative toxicity of the chemotherapy, drug resistance and the cancer recurrence present real challenges in treating ovarian cancer after using these classical treatments [4]. Therefore, an additional regime is needed to increase the therapeutics efficacy.

Several studies presented that tumor cells use many immunosuppressive mechanisms to restrict the antitumor immunity [5, 6]. Tumor cells adaptive mechanism includes induced expression of checkpoint molecule or programmed death-ligand 1 (PD-L1) [7]. The binding of the ligand PD-L1 with its corresponding receptor PD-1 leads to a suppression of anti-tumor immunity [8]. Consequently, this suppression is mediated through induction of T cell apoptosis and functional exhaustion in the tumor microenvironment [9]. Recent findings reveal that PD-L1/PD-1 activation requires myeloid cells for their antitumor immunity suppression [5]. This suppression is blocked by anti-PD-L1 and anti-PD-1 antibodies, which are used as treatment in many types of cancer.

Despite the prognostic value of PD-L1 expression, the immunological pathway with high expressions of PD-L1 in ovarian cancer is rarely studied and the molecular mechanism that includes immune cells and tumor cells remain elusive [10]. Nowadays, immunotherapy clinical trials that attempt to block PD-1/ PD-L1 interactions are widely conducted with a reasonable overall response rate (19–25%) [11, 12]. Although these clinical trials showed promising results in tumor regression, but cancer relapse still occurs without any clear explanation, suggesting that, cancer recurrences correlate with previous PD-L1 expression and arises from cancer stem cells (CSCs). High levels of PD-L1 molecules were detected on the surface of cancer stem cells isolated from colon cancer and breast cancer whereas non- cancer stem cell isolated from the same tissues did not express PD-L1 [13]. Therefore, the expression of PD-L1 on the surface of stem cells may play a major role in chemotherapy resistance and induction of disease recurrence [14].

The existence of CSCs is considered one of the reasons for cancer relapse and metastases as they are resistant to most chemotherapy treatments [15]. CSCs causes tumor cell heterogeneity and are responsible for remission following therapy [16]. To fully reverse the trend, CSCs in malignancies must be treated. Various strategies like dendritic cells (DC), oncolytic viruses, adaptive T-cells, immunological checkpoints inhibitors drugs are now targeting CSC to treat various malignancies [17]. Stem-like cells expressing certain markers, especially CD44 are reported to be chemotherapy resistant and considered to cause poor survival in serous epithelial ovarian cancer [18, 19]. CSC with high expression of CD44 are also considered a poor prognostic indicator in different types of tissues such as colorectal cancer, breast cancer, and ovarian cancer tissues [20, 21]. Activation mechanisms of stem cells and its association with the prognosis of ovarian cancer is poorly understood. This study might bridge the gap in our understanding by exploring the role of predictive biomarkers for ovarian cancer. The aim of the study is to investigate the role of PD-L1 expression in tumor micro-environment cells including immune cells and CSCs. The findings are likely to help in improving knowledge about the potential benefits of ovarian cancer immunotherapy in certain subset of patients and risk factors for relapse and resistance that are mediated by CSCs.

## Materials and methods

### Patient cohort and sample collection

This retrospective study was approved by the Institutional Review Board (IRB) affiliated to King Fahad Specialist Hospital- Dammam and the informed consent was waived by the IRB for the retrospective patients’ cases under protocol # ONC0340. All procedures were performed in accordance with office of Human Research Protection guidelines at KFSH-D.

**Fig. 1:**
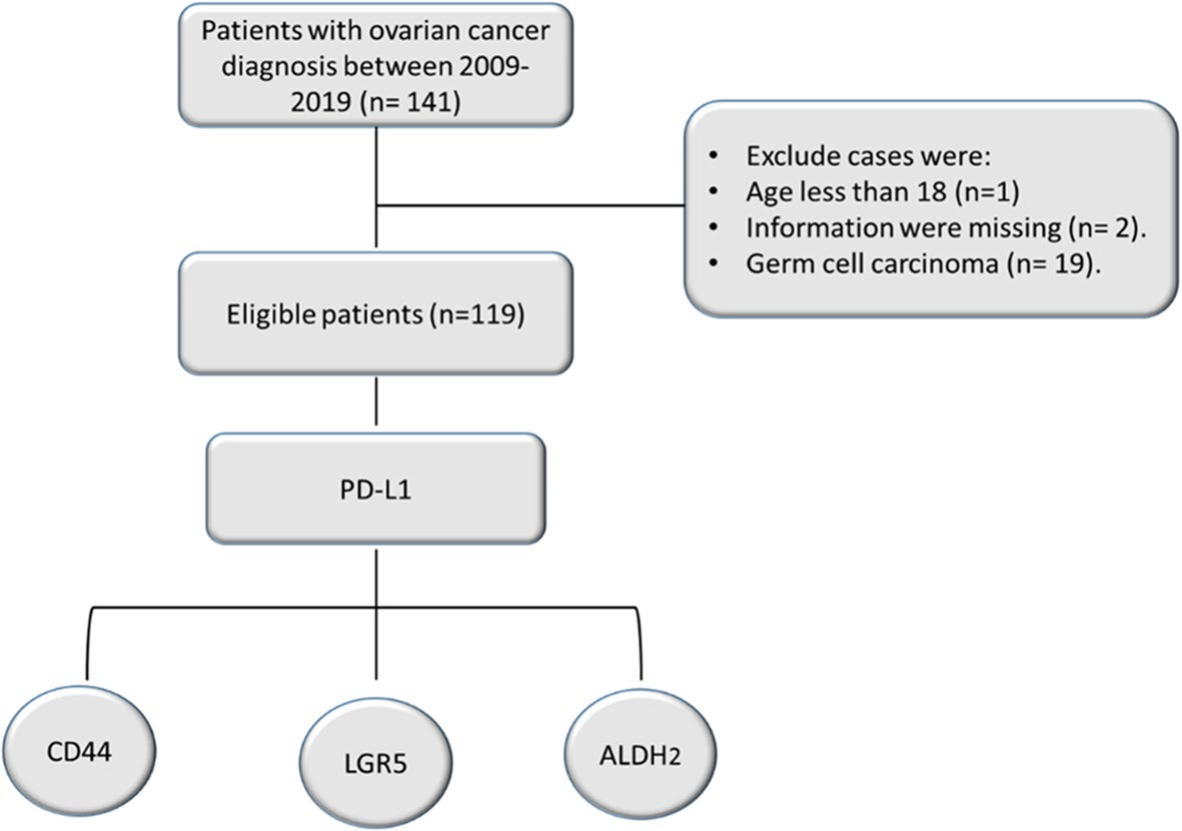
A flow chart diagram shows the ovarian cancer selection and the investigated protein expressions

A total of 141 ovarian cancer cases were extracted from the cancer registry at King Fahad Specialist Hospital- Dammam. The surgical tissues for 119 ovarian cases were eligible for histological experiments (Fig. 1). Specialized pathologists reviewed Hematoxylin and Eosin-stained sections to assess the ovarian cancer histological type according to the World Health Organization classification. Ovarian cancer staging assessments were done according to the Tumor-Node-Metastasis (TNM) and International Federation of Gynecology and Obstetrics (FIGO) classifications. Formalin-Fixed Paraffin-Embedded (FFPE) samples of ovarian cancer from different tumor cores were collected for immunohistochemistry experiments. Patients’ demographic and clinicopathological data were collected from the local cancer registry and patients’ medical record.

### Immunohistochemistry experiment (IHC)

FFPE samples were sectioned at 4 μm to perform several IHC experiments. IHC for PD-L1 was performed using a mouse monoclonal anti-PD-L1 antibody (PD-L1 IHC 22C3 pharmDx,) and DAKO auto Stainer system (DAKO, Denmark) following the manufacturer’s instructions. IHC for CD44 was performed using VENTANA BenchMark (Roche, Switzerland). Anti-CD44 (SP37) rabbit monoclonal antibody and iVIEW DAB detection kit (Roche, Switzerland) were used to detect CD44 expression following the manufacturers’ instructions.

To detect the expressions of tumor infiltrating lymphocytes and stem cell markers, manual IHC experiments were performed. Briefly, tumor sectioned were deparaffinized and rehydrated using xylene and graded ethanol. Antigen retrieval was performed by heating the tumor sections using epitope retrieval solution pH 9 and PT LINK pre-treatment system (Agilent, CA). All tumor sections were incubated with peroxidase inhibitor, blocked with Novocastra Protein Block solution (Leica Biosystems, Germany) and then incubated with primary antibodies.

For tumor infiltrating lymphocytes, Anti-CD8 (clone 4B11) mouse monoclonal antibody and Anti-CD4 (clone 4B12) mouse monoclonal antibody were used in a concentration of (1:100) (Leica Biosystems, Germany). For stem cell markers, anti LGR5 and anti ALDH2 antibodies (Invitrogen, CA) were used in concentration of (1:150) and (1:200), respectively. The protein expressions were detected using Novolink TM Max DAB (Polymer) kit (Leica Biosystems, Germany) following the manufacturers’ instructions. The immunoreactivity was visualized using M8 digital microscope and scanner, imaged and analyzed using Viewpoint Virtual Slide Viewing Software (Precipoint, Germany). Immunohistochemical protein expressions positivity was scored by defining the percentage of positive cells out of the whole tissue.

### Evaluation of the specimens

The tumor proportion score for PD-L1 membrane expressions were determined in the tumor tissues as follows; negative (No or less than 1%), weak (1–30%), moderate (40–60%) or strong (more than 60%). Thus, all sections were presenting more than 1% of PD-L1 were considered as PD-L1 positive and less than 1% were considered as PD-L1 negative. The TILs expressions, (CD8 and CD4) and stem cell marker expressions (CD44, LGR5, and ALDH2) in tumor tissues were considered as positive or high if the expressions are equal or more than 10% out of the tumor sections in three different random fields.

### Statistical analyses

Statistical analyses were performed using SAS statistical software (version 9.4) and IBM SPSS (version 29.0). We calculated means and proportions to report patients and tumor characteristics. The clinicopathologic variables’ statistical analysis was performed using Pearson’s Chi-squared or Fisher’s Exact test when appropriate. Survival functions for overall and cancer-free survival were generated for different ovarian histological types and PD-L1 expression using the Kaplan–Meier method. We used the Cox proportional hazard method to test the associations between PD-L1 expression and overall mortality. The model was adjusted for tumor characteristics (i.e., tumor size, stage, grade, and patient age).

## Results

### Ovarian cancer prevalence and PD-L1 expressions

**Fig. 2:**
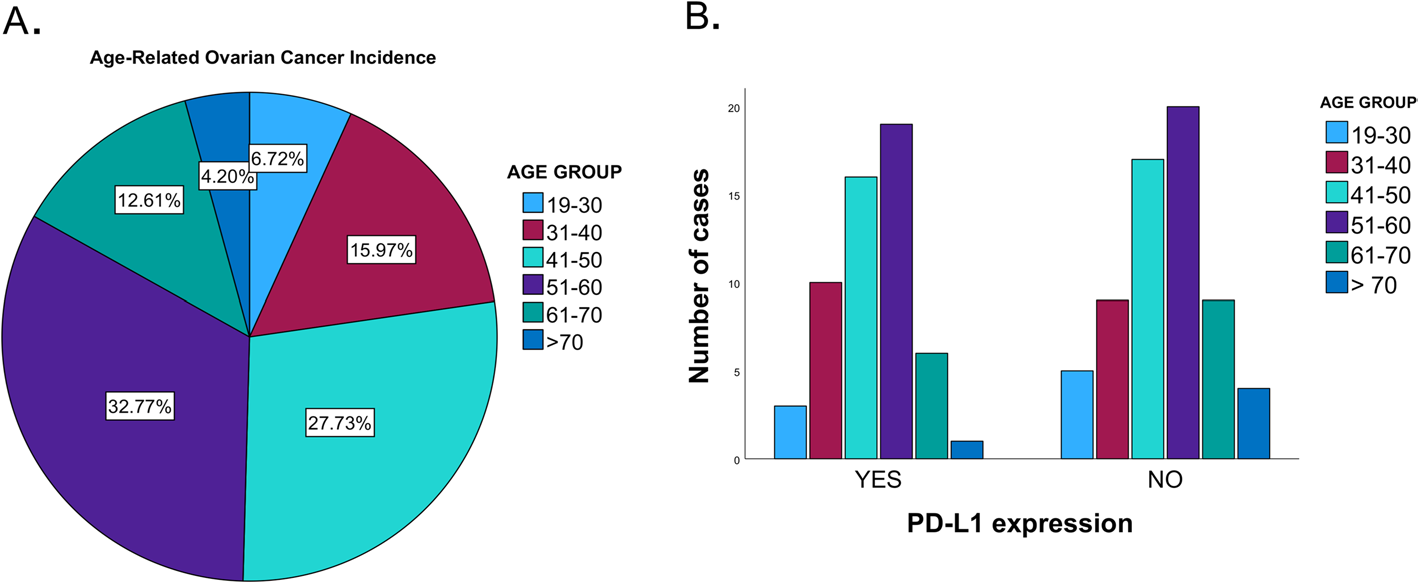
Prevalence of ovarian cancer and PD-L1 expressions in different age groups. **A** A colored chart shows age-related ovarian cancer. The incidence of ovarian cancer increases by age to reach 60 years old and then the risk starts to decrease. **B** A paragraph represents ovarian cancer cases with positive and negative PD-L1 expressions in different age groups

Our results from 119 eligible ovarian cancer patients found that aging is positively associated with the prevalence and incidence of ovarian cancer. The highest prevalence (33%) was in the women of 51 to 60 years old. Incidence was found to be less in younger women (Fig. 2A). The expressions of PD-L1 were found in all age groups and there is no statistically significant differences between PD-L1 positive and PD-L1 negative group in respect to the age (*p* = 0.8). Although women aged 51–60 years had the highest case numbers among PD-L1 positive group (*n* = 19, 48.7%), the similar levels also were shown in this age group of PD-L1 negative group (*n* = 20, 51%), (Fig. 2B) and (Table 1).

**Table 1:** Association of PD-L1 expressions and the Clinicopathological data of ovarian cancer patients

	Sample n (%)	PD-L1 n (%)		***p***-value
		No	Yes	
**Age at diagnosis**				
19–30	8 (6.72)	5 (62.50)	3 (37.50)	0.7475
31–40	19 (15.97)	8 (42.11)	11 (57.89)	
41–50	33 (27.73)	17 (51.52)	16 (48.48)	
51–60	39 (32.41)	20 (51.28)	19 (48.72)	
61–70	15 (12.61)	8 (53.33)	7 (46.67)	
≥ 71	5 (4.20)	4(80.00)	1 (20.00)	
**Tumor histological type**				
Serous carcinoma	74 (62.18)	36 (48.65)	38 (51.35)	0.6164
Endometroid carcinoma	14 (11.76)	7 (50.00)	7 (50.00)	
Clear cell carcinoma	8 (6.72)	6 (75.00)	2 (25.00)	
Mucinous carcinoma	13 (10.92)	7 (53.85)	6 (46.15)	
Granulosa cell carcinoma	7 (5.88	5 (71.43)	2 (28.57)	
Other	3 (2.52)	1 (33.33)	2 (66.67)	
**Tumor stage** ^**a**^				
Stage I	37 (36.27)	21 (56.76)	16 (42.24)	0.5131
Stage II	11 (10.78)	4 (36.36)	7 (63.64)	
Stage III	32 (31.37)	17 (53.13)	15 (46.88)	
Stage IV	22 (21.57)	14 (63.64)	8 (36.36)	
**Tumor grade** ^**b**^				
Low-grade serous carcinoma	11 (9.82)	5 (45.45)	6 (54.55)	0.7111
High-grade serous carcinoma	60 (53.57)	29 (48.33)	31 (51.67)	
Low-grade other carcinomas	25 (22.32)	14 (56.00)	11 (44.00)	
High-grade other carcinomas	16 (14.29)	10 (62.50)	6 (37.50)	
**Tumor size m(SD)** ^**c**^	12.23 (8.31)	13.57 (9.47)	10.86 (6.76)	0.1093

^a^A total of 17 observations were missing

^b^A total of 7 observations were missing

^c^A total of 22 observations were missing

### PD-L1 expression in different histological types of ovarian cancer

**Fig. 3:**
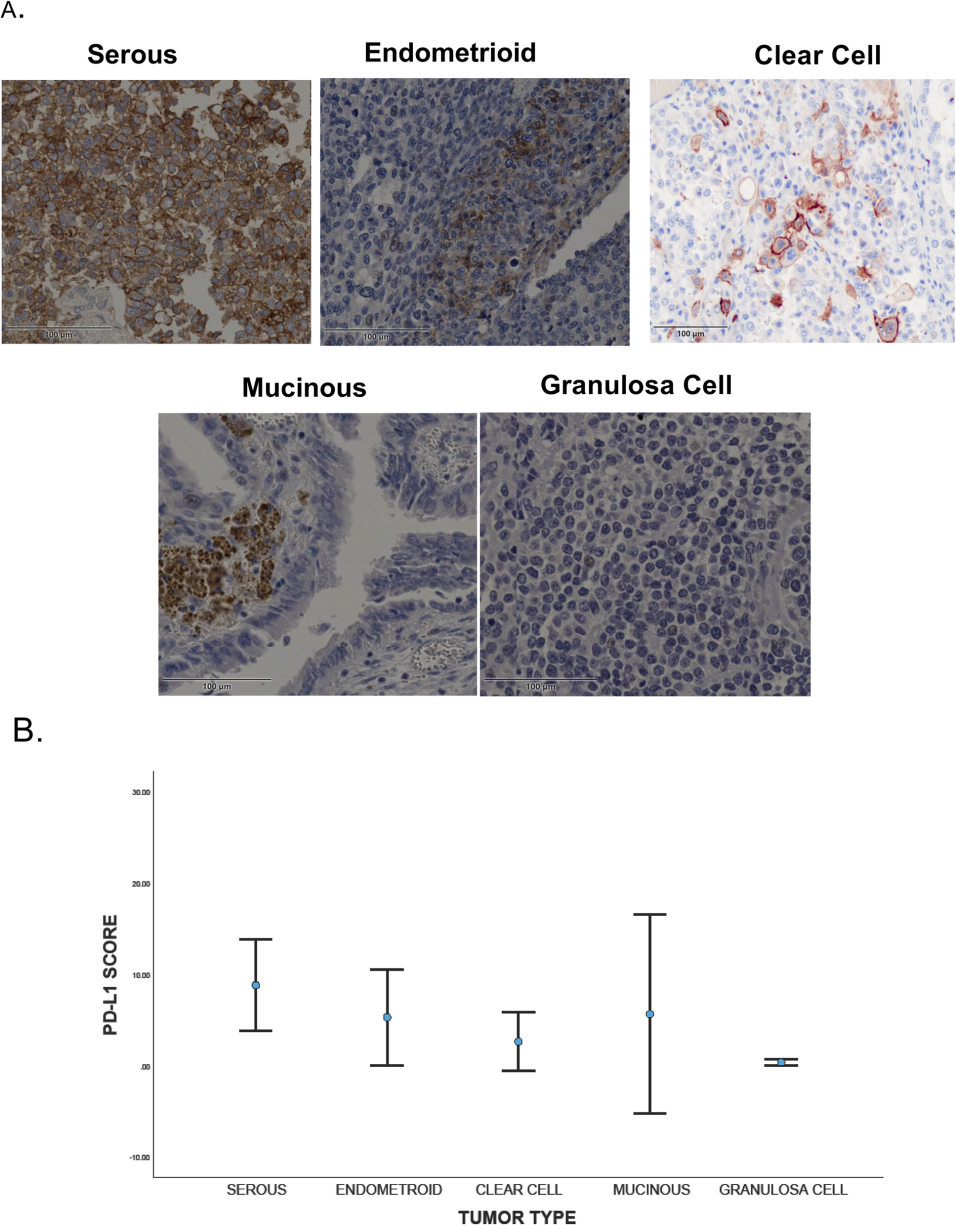
PD-L1 expressions in ovarian cancer. **A** Immunohistochemistry staining for human ovarian cancer shows different levels of PD-L1 expressions in different types of ovarian cancers. Scale bar is 100 μm. **B** Quntifecation analysis for PD-L1 expressions in different types of ovarain cncer shows the mean of PD-L1 scores for each type of ovarian cancer

The immunohistochemistry experiments for all histological types of ovarian cancer presented diverse levels of PD-L1 expressions in the tumor tissues (Fig. 3A). PD-L1 protein expressions have been detected in 47.8% of the ovarian cancer patients. This occurred for different types of ovarian cancer comprising the serous carcinoma, endometrioid, mucinous, granulosa cell, and clear cell carcinoma. Our result showed that serous carcinoma (51.35%) and endometroid carcinoma (50%) tissues presented the highest numbers of PD-L1 positive cases. The mucinous, granulosa cell and clear cell carcinoma presented 46, 28, and 25%, respectively (Table 1). However, the quantification analysis for PD-L1 expressions in different types of ovarian cancer showed the highest levels of expressions in serous cancer tissues (Fig. 3B).

### PD-L1 expression is associated with a better prognosis in ovarian cancer

**Fig. 4:**
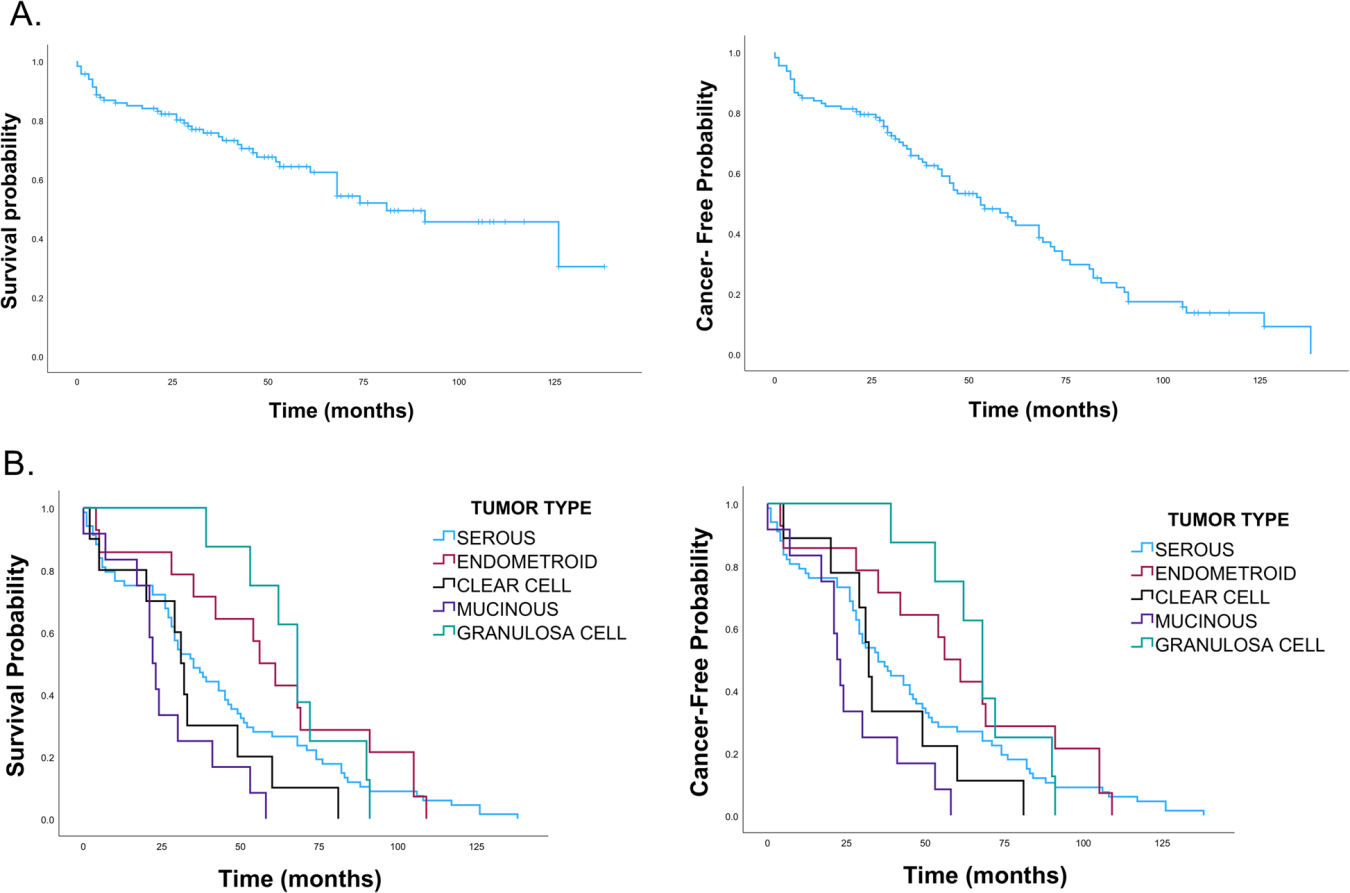
Kaplan-Meier survival curves of epithelial ovarian cancer. **A** The upper panels show ten - year survival probability and the cancer-free probability for all epithelial ovarian cancer. **B** The lower panels show the ten - years survival probability and the cancer-free probability for different histological types of ovarian cancer. The curves demonstrate the decreased survival and cancer-free probabilities rate for clear cell carcinoma and mucinous cancer compared to other types of ovarian cancer

The survival probability and cancer-free probability were significantly decreased after 20 months from the time of patient’s diagnosis for all ovarian cancer histological types. However, clear cell and mucinous carcinomas had the poor survival probability compared to other ovarian cancer histological types with hazard ratio of 2.2 and 2.9, respectively (Fig. 4) and (Table 2).

**Table 2:** Five-year survival probability for different ovarian cancer histological types

Histological type	Survival Probability (95% CI)				
	1-year	2-year	3-year	4-year	5-year
All	94.4 (88.1–97.5)	89.2 (81.3–93.9)	69.1 (58.6–77.5)	60.5 (49.5–69.8)	48.4 (37.3–58.6)
Clear cell carcinoma	84.6 (30.5–97.7)	67.7 (20.5–90.8)	33.8 (4.7–68.1)	33.8 (4.7–68.1)	33.8 (4.7–68.1)
Endometroid carcinoma	92.6 (57.9–98.9)	92.6 (57.9–98.9)	77.2 (44.6–92.0)	77.2 (44.6–92.0)	60.0 (28.7–81.2)
Mucinous carcinoma	100.0 (100.0–100.0)	66.7 (28.2–87.8)	40.0 (9.8–69.7)	26.7 (4.1–57.9)	8.9 (0.1–43.0)
Granulosa cell carcinoma	100.0 (100.0–100.0)	100.0 (100.0–100.0)	100.0 (100.0–100.0)	85.7 (33.4–97.9)	71.4 (25.8–92.0)
Serous carcinoma	93.9 (84.7–97.7)	92.2 (82.3–96.7)	69.6 (55.6–80.0)	60.8 (46.1–72.6)	48.4 (33.5–61.8)
Other	100.0 (100.0–100.0)	100.0 (100.0–100.0)	100.0 (100.0–100.0)	66.7 (5.4–94.5)	66.7 (5.4–94.5)

**Fig. 5:**
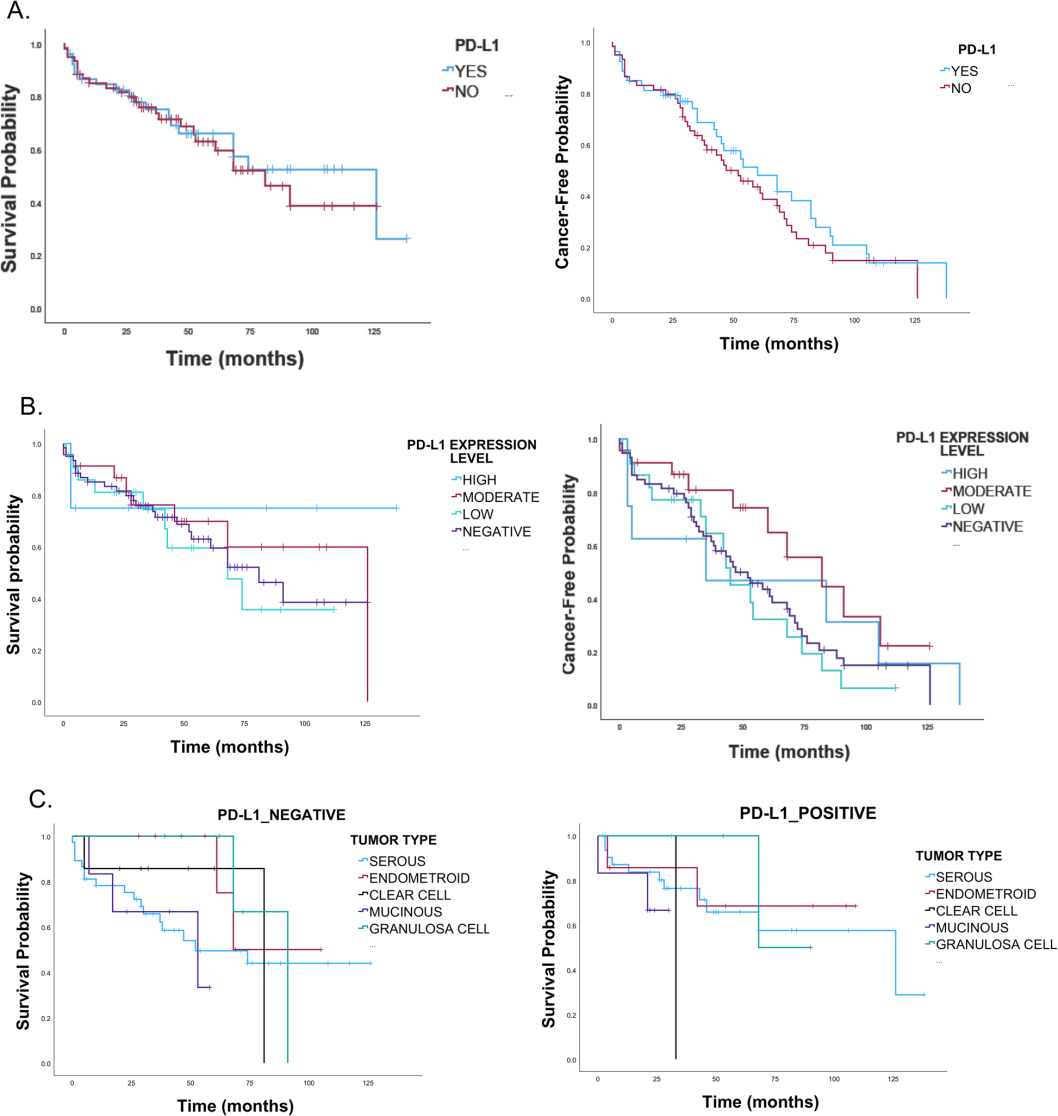
Kaplan-Meier curve of survival probability and cancer-free probability for ovarian cancer with and without PD-L1 expressions. **A** Upper panels show the survival probability and cancer-free probability for epithelial ovarian cancers with and without PD-L1 expression. The overall analysis shows that there is no significant difference between PD-L1 positive and PD-L1 negative. **B** Middle panels show the survival probability and the cancer-free probability for different levels of PD-L1 expressions in epithelial ovarian cancer. **C** Lower panels show survival curves for different types of ovarain cancer with absence of PD-L1 expressions (left) and positive expressions (right). The survival curves do not show a signfecant difference between PD-L1 negtive and PD-L1 positive

Amongst all histological types of ovarian cancer, patient with positive expressions of PD-L1 had slightly better cancer-free survival rate compared to patients with no PD-L1 expressions (HR 1.6, 95% CI 0.5, *P* < 0.06), (Fig. 5A). The overall 10-year death rate analysis for the PD-L1 positive and PD-L1 negative had no difference with good prognosis for all ovarian cancer patients. Nevertheless, ovarian cancer patients with moderate expressions of PD-L1 have better cancer-free probability compared to PD-L1 negative patients (Fig. 5). However, the survival for different types of ovarian cancer did not present significant differences in PD-L1 positive group or in the PD-L1 negative group (Fig. 5C). This might indicate the minimum role of PD-L1 on the ovarian cancer prognosis.

### Tumor infiltrating lymphocytes (TILs) expressions in ovarian cancer patients associated with PD-L1 expression

**Fig. 6:**
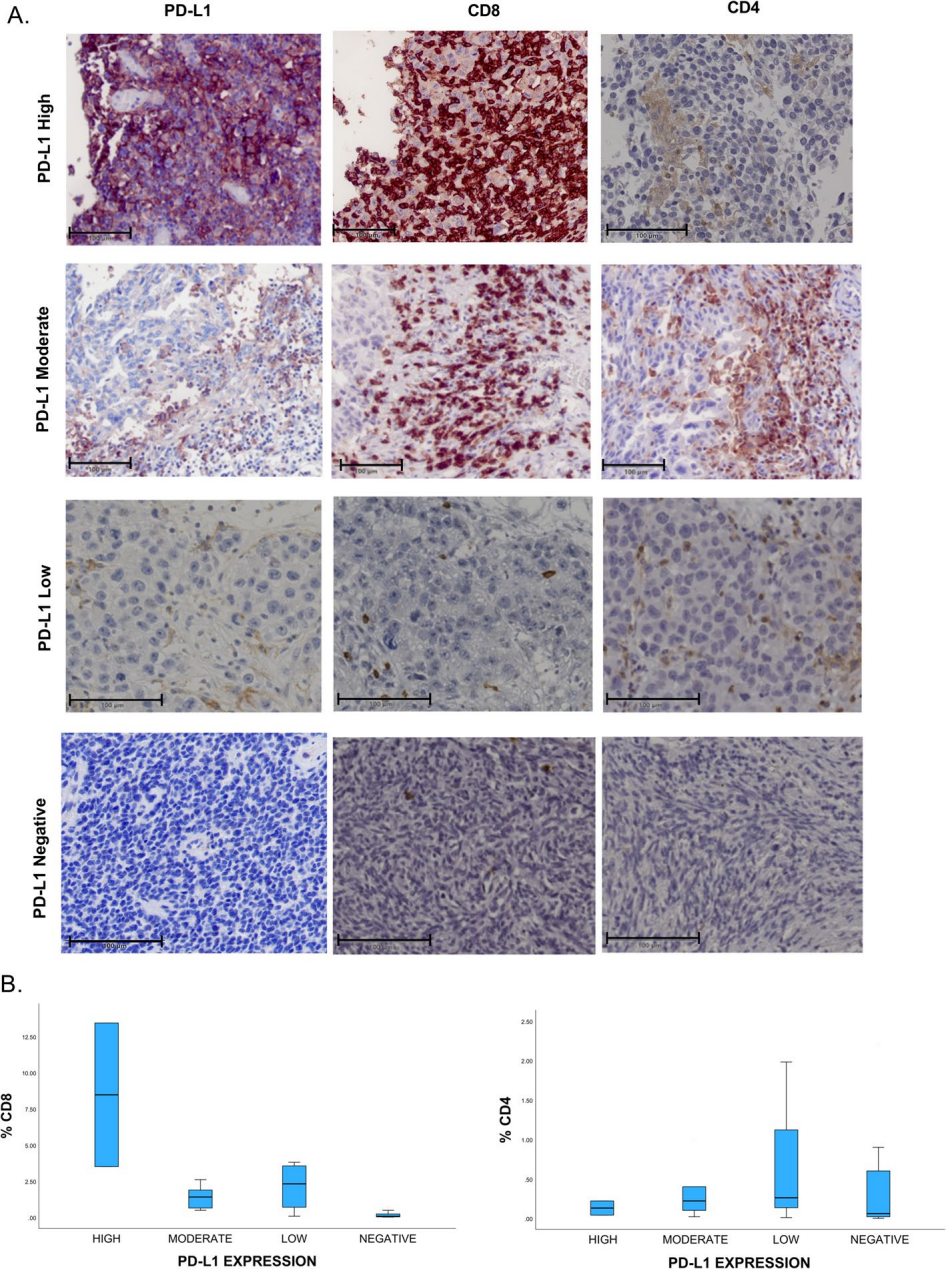
Immunohistochemistry experiment for PD-L1 and tumor infiltrating lymphocytes. **A** Immunohistochemistry staining show the tumor infiltrating lymphocytes expressing CD8 and CD4 in ovarian cancer patients with different levels of PD-L1 expressions. Strong expressions of CD8 and moderate expression of CD4 in the cancer tissues with high expressions of PD-L1. Scale bar is 100 μm. **B** Quntifecation analysis for CD8 and CD4 expressions in different PD-L1 expressions group

The levels of lymphocyte infiltration were assessed in the ovarian cancer tissues by staining the cancer sections with antibodies against lymphocyte markers CD8 and CD4. Serial sections from the tumor were incubated first with PD-L1 antibodies to confirm the expressions of PD-L1 and then with CD4 and CD8 antibodies. We found that the ovarian cancer cells that expressed PD-L1 proteins on their membrane also expressed very high expressions of CD8 and moderate expressions of CD4 in all ovarian cancer histological types (Fig. 6A and B). TILs expressions in all ovarian cancer cases showed significant frequencies in PD-L1 positive groups compared to PD-L1 negative groups (Table 3).

**Table 3:** Association of PD-L1 with Tumor infiltrating lymphocytes expressions

PD-L1	CD8 n (%)			CD4 n (%)		
	High	Low/Negative	*p*-value	High	Low/Negative	*p*-value
Yes	9 (81.8)	2 (18.18)	0.0047	9 (81.8)	2 (18.18)	0.0017
No	5 (27.7)	13 (72)		4 (2.2)	14 (77.7)	

### High expression of PD-L1 on ovarian cancer is associated with stem cells expressions

**Fig. 7:**
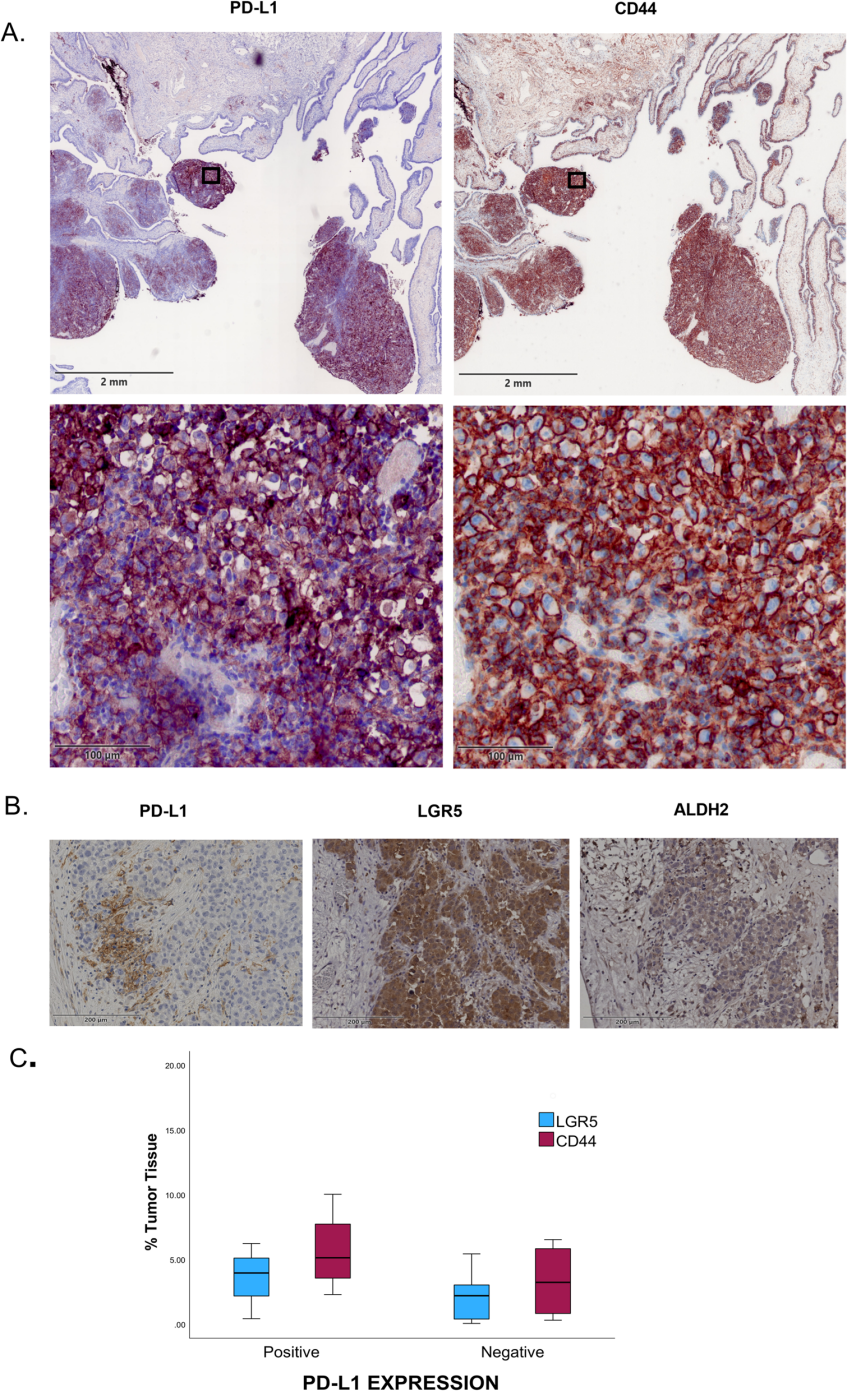
Immunohistochemistry for stem cell biomarkers in ovarian cancer. **A** Immunohistochemistry images for ovarian cancer tissues show positive expressions of PD-L1 and colocalize expressions of PD-L1 and the stem cell marker CD44. **B** Serial sections for ovarain cancer show PD-L1 expressions and stem cell markers expressions (LGR5 and ALDH2). **C** Quntfication analysis for the stem cell marker expressions in positive and negative PD-L1 tissues

**Fig. 8:**
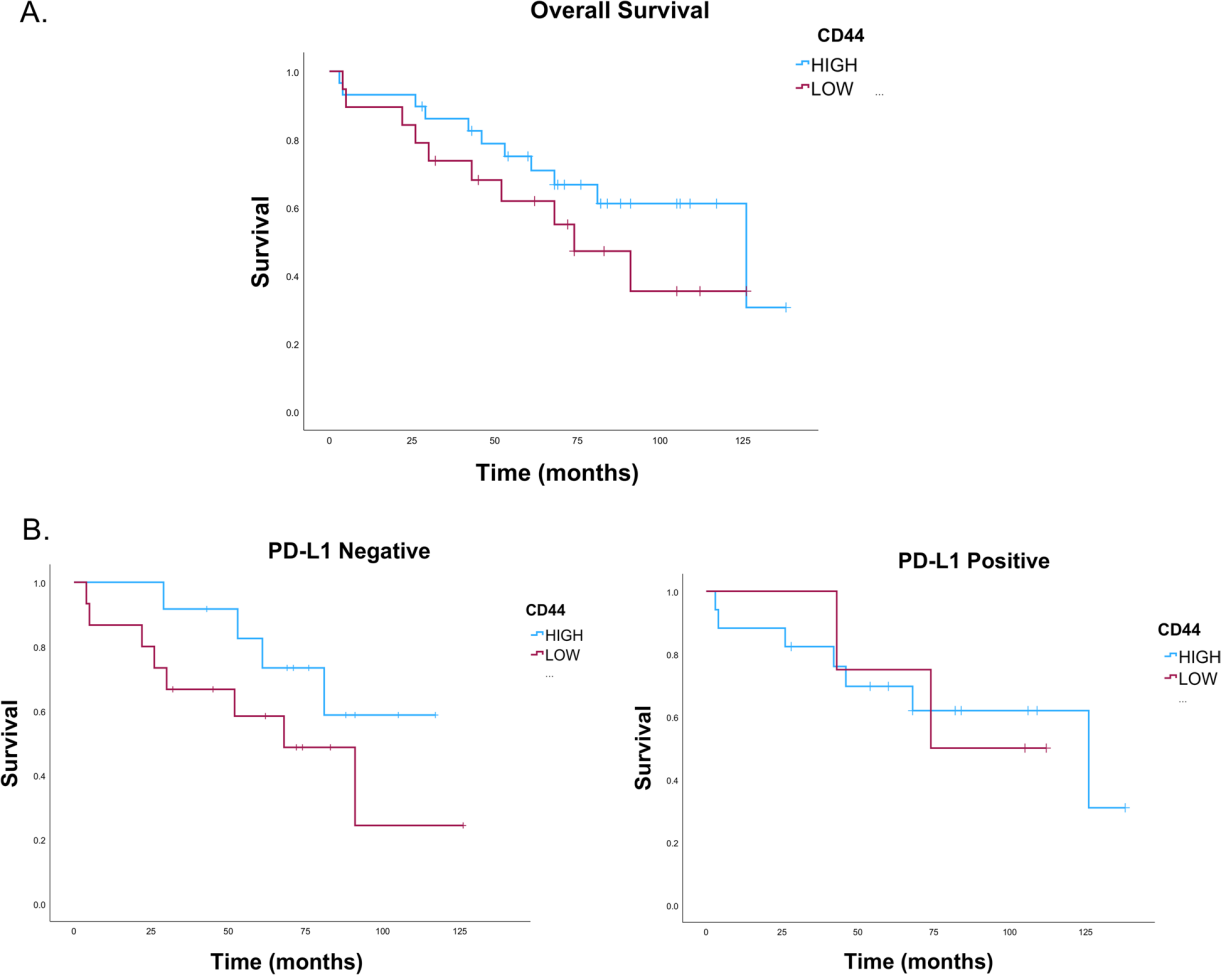
Kaplan-Meier curve of survival probability for ovarian cancer with and without CD44 expressions. **A** Upper panel presents the survival probability for all ovarian cancers expressing CD44 that show a better survival of ovarain cancer patients with high expressions of CD44. **B** Lower panels present the survival probability for ovarian cancers expressing CD44 and PD-L1 negative (left) or PD-L1 positive (right). The survival analysis indecates a better prognosis for ovarain cacer patients that expressing CD44 asscocited with absence of PD-L1 expressions

The immunohistochemistry staining for several stem cell markers showed different levels of proteins expressions on the cancer cells membrane. We found that Stem cell markers CD44 and LGR5 are highly expressed by serous cancer (*P* = 0.006 and *P* = 0.6, respectively) (Table 4). Similarly, high PD-L1 expression was also associated with serous cancer. Consequently, the high expression of CD44 was 77.2% associated with positive expression of PD-L1 and LGR5 was 100% associated with positive expression of PD-L1. Whereas other stem cell markers (ALDH2) showed no high frequency in PD-L1 positive compared to PD-L1 negative group (Table 5). Although the serial sections of different types of ovarian cancers showed the expressions of PD-L1 and the stem cell markers on the same section, the expressions of CD44 and LGR5 were colocalized with PD-L1 expressions (Fig. 7A&B). The quantification analysis for the stem cell markers showed significant upregulation of CD44 expressions and slight increase of LGR5 expressions in PD-L1 positive group compared to PD-L1 negative group (Fig. 7C). In addition, our survival analysis showed that CD44 expression is slightly associated with a better prognosis for ovarian cancer cases and for the PD-L1 negative ovarian cancer (HR = 1.6, 95% CI [0.7–3.7] and (HR = 2.4, 95% CI. [0.74–7.9]), respectively. However, the association of CD44 and PD-L1 expressions in PD-L1 positive group decreased the survival compared to CD44 negative (HR = 0.8, 95% CI [1–6.7]), (Fig. 8).

**Table 4:** Frequencies of stem cell expressing CD44 and LGR5 markers by histological type

Histological type	CD44 ^High^	CD44 ^Low/Negative^	*p*-value	LGR5 ^High^	LGR5 ^Low/Negative^	*p*-value
Clear cell carcinoma	1	0	0.006	1	0	0.6878
Endometroid carcinoma	5	0		3	1	
Mucinous carcinoma	1	0		0	1	
Serous carcinoma	14	4		8	3	
Other	0	4		2	0

**Table 5:** Association of PD-L1 with stem cell expression

PD-L1	CD44 n (%)			LGR5 n (%)			ALDH2 n (%)		
	Yes	No	*p*-value	Yes	No	*p*-value	Yes	No	*p*-value
Yes	17 (77.2)	5 (22.7)	0.04	5 (100)	0(0)	0.25	4 (57)	3 (43)	1.000
No	12 (44.4)	15 (55.5)		9 (64.2)	5(35.7)		12(63)	7 (37)

## Discussion

Ovarian cancer is among the cancers with the highest mortality after the breast cancer in Saudi women [22]. Most of patients would initially benefit from surgery and chemotherapy, but recurrences are reported in more than  80% of patients with advanced ovarian cancer [23]. Cancer metastases and recurrences are usually associated with poor prognosis that develops in patients with impaired immunological system that induces the treatment resistance [24]. Cancer heterogeneity and tumor microenvironment plays significant role in cancer resistance [25, 26]. Therefore, it is important to understand the immune milieu of the disease and the role of cancer cells and other types of cells that regulates the immune response and cancer treatment. Accumulating evidence demonstrated the role of PD-L1 expression in patients’ survival and treatment response [6, 10, 27–31] but the role of PD-L1 / PD-1 in cancer initiation or recurrence is not known yet.

In our study, we investigated PD-L1 expressions in ovarian cancer and its association with specific pathological and clinical outcomes, and the tumor microenvironment that include lymphocytes and cancer stem cell populations. PD-L1 expressions were investigated among different age groups of ovarian cancer patients. Our finding revealed the positive correlation of the ovarian cancer incidence with patients’ age, which is consistent with previous reports. The highest prevalence of ovarian cancer in the middle age group is similar to other studies [10]. Consequently, PD-L1 expressions were detected largely in the middle-aged ovarian cancer patients. This could be explained by the fact that most middle-aged patients present with high-grade cancers, which is associated with high PD-L1 expression. Moreover, these results indicates that there is no significant association of PD-L1 expressions with early onset of ovarian cancers or predisposition that disregards the risk factor of inherited genetic mutation.

In addition, we demonstrated PD-L1 expressions in different histological types of ovarian cancer. Our result showed that considerable percentage of ovarian tumors expressed PD-L1 on the surface of the cancer cells. These findings are in consistent with previous reports that presented the association of PD-L1 expressions with different types of ovarian cancer [32]. However, PD-L1 expression was shown to be inconsistent between original epithelial ovarian cancer and highly associated with peritoneal metastases [32]. Therefore, testing PD-L1 as a possible biomarker for predicting response to anti–PD–L1 therapy may necessitate an examination of associated metastatic lesions [32].

Our study further highlighted the ovarian cancer clinicopathological characteristics and its association with PD-L1 expression. We found that the survival probability and cancer free probability for ovarian cancer expressing all levels of PD-L1 expressions presented no significant differences compared to negative PD-L1 expressions. However, categorizing the levels of PD-L1 expressions demonstrated that the ovarian cancer patients that presented with moderate PD-L1 expressions have slightly better cancer-free probability compared to PD-L1 negative, although the high PD-L1 expression was reported previously to be associated with favorable prognosis in ovarian cancer [33]. Like other studies, we have found significant association of high PD-L1 expressions with serous carcinoma [34]. Contrary to other studies, we found that PD-L1 expressions are not related to the tumor stage or grade as reported previously [33]. However, the abundant expressions of PD-L1 in high grade ovarian cancer was reported only in peritoneal metastasis but not in primary tumor [32] which might be the reason for this contrast.

Association of high PD-L1 expression with shorter survival is well known in breast cancer but not in ovarian cancer [35–37]. However, one study suggested that PD-L1 overexpression in endometroid adenocarcinoma cancer is significantly decreases cancer cell invasion and migration in vitro and associated with favorable survival for endometroid cancer patients in vivo [38]. This finding may explain the slight increase of the overall survival for ovarian cancer patients in our study.

Although the association of PD-L1 with ovarian cancer survival is controversial, our findings have been supported by previous research studies and meta-analysis study [37]. In consistent with our results, Mills’ et al. in 2018 [39] also reported no relationship of PD-L1 expressions with overall survival in ovarian cancer. Whereas the meta-analysis studying 1228 patients with ovarian cancer reported a positive relationship of PD-L1 with worse progress-free survival [40]. Several factors might affect PD-L1 expressions and the survival for ovarian cancer patients. The role of estrogen and its receptors (ER) have been reported to regulates the tumor immunity in breast cancer [41]. However, it has been shown that high PD-L1 expression was associated with ER-negative breast cancer. Further, ER receptors decreases PD-L1 expressions through downregulating IL-17 signaling [42].

In ovarian cancer, although some studies found that estrogen is enhancing the ovarian cancer proliferation and tumor growth, other studies found that high expressions of estrogen receptors are associated with better prognosis and progression free survival. PD-L1 expressions were expressed in endometroid cancer that have negative expressions of estrogen and progesterone [43]. Thus, hormone receptors might have a role on PD-L1 expressions, treatment response and survival for ovarian cancer patients.

Further investigation of estragon receptors and the association of PD-L1 expression in ovarian cancer are needed. The crucial information that will be provided by the future studies might help in better understanding of the ovarian cancer microenvironment. Accordingly, determining the treatment management for ovarian cancer patients using hormonal and immune therapy. To demonstrate the tumor microenvironment, we found that the positive expressions of PD-L1 on ovarian cancer cells were significantly associated with high percentage of TILs that express CD8 and CD4. High rate of lymphocytes infiltration might be a result to the high expression of PD-L1, which act as ligands to the lymphocytes expressing PD-L1. High tumor lymphocytes infiltration is known to be favorable prognostic factor in ovarian cancer. However, the association of TILs with PD-L1 might increase the drug response. Due to the study limitations, we were not able to investigate PD-1 expressions on TILs. The other way around, the presence of the lymphocytes may induce PD-L1 expressions in the tumor through IFN-γ signaling [44].

Numerous studies including our study investigated CD8+ and CD4+ to detect lymphocytes trafficking to the tumor to determine the association with PD-L1 expressions or with the disease prognosis, but activation status of these TILs are infrequently studied [45–50]. For example, high expressions of either PD-1 and/ or PD-L1 in the tumor microenvironment is associated with reducing effector T cells and inducing the activity of T-regulatory cells (T-reg) which impairs the immune response [51]. Most of the studies detected the tumor infiltrating lymphocytes as prognostic factor regardless of the T cells activity. Therefore, determining the immune inhibitory activity of these cells in the tumor using specific T cells biomarkers like CD45RA and CD45RO expression on CD8+ T cells are very important to know the activation status [52, 53].

For further demonstration of the ovarian cancer microenvironment, we have investigated cancer stem cells expressing CD44, LGR5 and ALDH2. Cancer stem cells comprise an essential component of tumor cells population and are mainly associated with cancer initiation, progression, metastasis, and recurrence [18–21, 54–56]. The results of the recent study should attract special attention to other prognostic factors such as stem cells that presenting CD44 and whether the expressions were associated with high PD-L1 expressions or not. These potential prognostic biomarkers should be used not only for prognosis predication but also for therapeutic management and selecting the appropriate targeted therapy.

Although CD44 expression is associated with ovarian cancer patients, its role in the patients’ prognosis is controversial [57]. While a study found a strong association between CD44 expression and poor prognosis [58], other studies presented that CD44 associated with a better prognosis [59] whereas it is not an independent predicator prognosis biomarker of ovarian cancer [60]. Our study observed that CD44 were highly expressed by PD-L1 positive tissues and associated with favorable prognosis. These findings have been supported by several other studies [13, 59, 61]. Many authors found that cancer stem cells expressing CD44 in lung cancer tissues, breast and colorectal cancer cell lines have high expressions of PD-L1 on their surface compared to CD44 negative populations [13, 61]. In consistent with our findings, a study showed that invasive breast cancer that express high levels of PD-L1 expressions positively regulate cancer stem cells that express CD44 ^high^, CD24 ^low^ and OCT4^high^ [62]. Further, PD-L1 knockdown in breast cancer cells downregulated the expression of CD44 and upregulated the expression of CD24 [63], suggesting that PD-L1 expression positively regulates stem-like cells activity in the cancer tissues [56].

As with the majority of other research studies, this study was subjected to some limitations. The sample size for some histological types of ovarian cancer was insufficient for the statistical measurement and that could have an impact on the outcomes. Although we found that the clear cells and mucinous cells that expressing PD-L1 have poor prognosis compared to PD-L1 negative, this finding does not reflect the general populations of clear and mucinous ovarian cancer because of the small sample size. Further studies are needed to detect PD-L1 expressions in a bigger sample size for each histological type of ovarian cancer.

In addition, we found in our study that PD-L1 expression is highly associated with stem cell markers. However, several studies have proposed different stem cell markers that associated with cancer recurrence [19, 21]. Whereas in our study we were not able to find out if the high expression of stem cell markers is associated with disease recurrence. We anticipated that high PD-L1 expressions and stem cells population that express specific biomarkers might associate with the late recurrence. Therefore, multiple stem cell markers need to be determined in ovarian cancer that associated with PD-L1 expressions and long follow up for the patients that determine the late recurrence. We did not separately test the PD-L1 expressions in the peritoneal metastases and primary tumor of the same patient. Therefore, we were not able to distinguish the metastasis from primary tumor in our analysis. In addition, TILs trafficking the cancer tissue must be studied if they are effector, active or immune inhibitory T cells through using specific biomarkers for each stage of T cells [49, 64, 65].

Finally, the controversial result of the PD-L1 expression and the association with patients’ clinicopathological characteristics might be a consequence of the facts that the scoring system and the detection method of PD-L1 are varied among different studies. Therefore, we are strongly recommending further studies to unify the scoring system, identify PD-L1 positive cells (membranous or/and cytoplasmic) and include several surgical resected cancer tissues from the same patient to detect PD-L1 expressions [32, 66].

## Conclusions

Our study has demonstrated the association of PD-L1 expressions with ovarian cancer outcomes. PD-L1 expressions are positively associated with better prognosis in ovarian cancer and tumor infiltrating lymphocytes presenting CD8 and CD4. Additionally, we proposed that ovarian cancers expressing PD-L1 are highly associated with stem cells. The association of PD-L1 and cancer stem cells warrant further studies in ovarian cancer recurrence, which we believe will lead to the predictive biomarkers for ovarian cancer recurrence and developing a proper treatment strategy.

## Data Availability

The data is available upon a reasonable request from the first author (Kholoud Alwosaibai).
